# ChatGPT-generated rehabilitation programs in sports physiotherapy: an expert evaluation and a mixed-methods study of clinical applicability

**DOI:** 10.3389/fmed.2026.1935702

**Published:** 2026-08-26

**Authors:** Adem Cali, Mehmet Erdem Yorukoglu, Gorkem Acar, Ertugrul Safran

**Affiliations:** 1 Faculty of Health Sciences, Department of Occupational Therapy and Rehabilitation, Istanbul Topkapi University, Istanbul, Türkiye; 2 Dr. Savas Kudas Clinic, Ankara, Türkiye; 3 Faculty of Sports Sciences, Istanbul Gelişim University, Istanbul, Türkiye; 4 Department of Physical Therapy and Rehabilitation, Faculty of Health Sciences, Istanbul Gelişim University, Istanbul, Türkiye

**Keywords:** artificial intelligence, ChatGPT, clinical decision support, expert evaluation, intraclass correlation coefficient, large language models, precision medicine, precision rehabilitation

## Abstract

**Background:**

Individualized rehabilitation supports safe return to sport, yet clinical workload may limit personalized program design. This study evaluated ChatGPT-4.1-generated programs for five sports injury scenarios using a structured rubric scored by two independent sports physiotherapy experts and examined inter-rater reliability and clinical applicability.

**Methods:**

Mixed-methods design combining rubric scoring with deductive qualitative content analysis of expert commentary. Five cases covered muscle (hamstring strain), ligament (anterior cruciate ligament [ACL] reconstruction), tendon (rotator cuff tendinopathy), neural (lumbar disc herniation) and bone (clavicle fracture) injuries. Exactly one 6-week tabular program per case was generated with ChatGPT-4.1 from a standardized prompt in a fresh session with memory, cross-chat history and custom instructions disabled. Two sports physiotherapists (>20 years’ experience) independently rated accuracy, exercise selection, progression and applicability on 1–5 scales, blinded to each other’s ratings, the verbatim prompt (including the imposed six-week horizon), model identity and study aims, while retaining the clinical scenario. ICC[2,1] (two-way random-effects, absolute agreement) with 95% CIs was computed across the 20 case × criterion units; exact and within-one-point agreement, weighted *κ* and Bland–Altman limits were supplementary.

**Results:**

The overall mean of 40 ratings was 3.85 ± 1.21 (95% CI 3.48–4.22), reported alongside disaggregated case- and criterion-level values. Case 5 (clavicle fracture) scored highest (5.00 ± 0.00), Case 2 (ACL) lowest (1.88 ± 0.83, 95% CI 1.18–2.57); exercise selection scored highest (4.20 ± 1.32, 95% CI 3.26–5.14), clinical applicability lowest (3.60 ± 1.26, 95% CI 2.70–4.50). Inter-rater reliability was good (ICC[2,1] = 0.84, 95% CI 0.52–0.94; *p* < 0.001); exact agreement 65%, within-one-point agreement 95%, weighted *κ* = 0.69. Experts praised structural organization and progression logic but noted weakness in multifactorial, postoperative-timing-sensitive cases.

**Conclusion:**

ChatGPT-4.1 generates plausible, structured programs for linear, protocol-based recovery (e.g., post-fracture), but performance declines markedly in complex, postoperative-staging-sensitive cases; it should serve as a clinician-supervised support tool, not an autonomous decision-maker. Because the six-week length was fixed across all scenarios, case complexity and prompt–scenario congruence are confounded. Findings rest on five programs from one prompting strategy and model, requiring confirmation in larger multi-prompt, multi-model studies.

## 1 Introduction

Personalized rehabilitation programs are a cornerstone of effective recovery in sports medicine and physiotherapy, supporting functional recovery, injury prevention, and safe return-to-sport outcomes (1, 2). Traditional rehabilitation planning relies heavily on clinical expertise, comprehensive assessment, and iterative adjustments based on the patient’s progress and response to treatment (1). However, in busy clinical settings, time constraints, variability in clinician experience, and workload pressures may limit comprehensive personalization and the timely adaptation of rehabilitation programs (3).

Artificial intelligence (AI), particularly large language models (LLMs) such as ChatGPT, has emerged as a promising approach for supporting rehabilitation program design and clinical decision-making (4–6). These generative models can process large amounts of data, synthesize evidence-based guidelines, and generate structured treatment recommendations tailored to individual patient characteristics (5). In recent years, AI-based solutions have been increasingly applied across various areas of healthcare, demonstrating potential in core components of current rehabilitation practice such as personalized care, outcome prediction, and real-time patient monitoring (2).

Among these technologies, LLMs such as OpenAI’s ChatGPT can rapidly integrate clinical information based on user input and generate structured rehabilitation recommendations, offering a scalable tool that may complement clinician expertise in treatment planning (7). Emerging evidence in physiotherapy and sports medicine supports this potential. For example, in a study evaluating ChatGPT’s alignment with clinical practice guidelines for musculoskeletal rehabilitation, the mean accuracy score was 4.62/5; while a very high level of agreement was observed for the disease-information category (weighted kappa = 0.90), agreement was lower for the rehabilitation-recommendation category (weighted kappa = 0.56), pointing to ChatGPT’s role as an auxiliary clinical decision-support tool (8). Similarly, in the context of sports science, resistance-training programs generated by different versions of ChatGPT (ChatGPT-3.5, ChatGPT-4o, and ChatGPT-4.1) have been evaluated by expert panels, showing that newer model versions (e.g., ChatGPT-4.1) tend to produce more detailed, consistent, and scientifically grounded recommendations than earlier versions (9, 10). A recent systematic review in orthopaedic sports surgery similarly demonstrated that ChatGPT responses show marked variability in accuracy, quality, and readability, with notable limitations in source attribution and readability level (11). A comprehensive scoping review on the use of LLMs in exercise prescription and physical activity recommendations has categorized applications in this field and highlighted research gaps that need to be addressed for clinical integration (12). Conceptual appraisals specific to physiotherapy have likewise emphasized that LLMs have the potential to reduce administrative workload and support patient education, but that clinician oversight remains indispensable with respect to accuracy, safety, and individualization (13, 14). In line with this trend, recent studies evaluating the musculoskeletal clinical-reasoning performance of LLMs in physiotherapy education, and examining physiotherapy students’ acceptance of AI-based chatbots, indicate that interest in integrating these technologies into education and clinical practice is growing rapidly (15, 16). Comparative work in musculoskeletal and orthopaedic settings has begun to benchmark different systems against one another and against clinical guidelines: ChatGPT-4o and DeepSeek AI have been compared for concordance with AAOS recommendations in clavicle fracture management (17); ChatGPT, Claude and Perplexity have been contrasted in a septic arthritis scenario, with the three systems differing significantly in content depth, unnecessary elaboration and reference support (18); and ChatGPT-5 and DeepSeek V3 have been evaluated for artificial intelligence-assisted patient education in foot and ankle disorders (19). These studies consistently show that performance differs both between models and between task types, which frames the single-model design of the present study as a deliberate first step rather than a comprehensive benchmark.

Despite the growing interest in AI-assisted rehabilitation, research systematically evaluating LLM-generated rehabilitation prescriptions in the context of sports rehabilitation - particularly in conjunction with structured expert-evaluation processes - remains relatively scarce. The present study is not designed to address cross-version or cross-model comparison, and no gap of that kind is claimed here. It differs from the closest existing work — expert-panel evaluation of ChatGPT-generated resistance-training programs (9) — in three specific respects: the setting is clinical rehabilitation after injury rather than performance training; the scenarios sample systematically across five distinct injured tissue types (muscle, ligament, tendon, neural and bone) rather than a single task domain; and the rubric separates scientific accuracy, exercise selection, progression logic and clinical applicability into distinct domains, so that the profile of strengths and weaknesses within one program can be described rather than summarised as a single quality score. The extent to which AI outputs align with clinician judgment in this setting requires further research to support safe and effective clinical integration.

Precision medicine in sports rehabilitation aims to tailor rehabilitation strategies according to each patient’s biological, functional, and clinical characteristics rather than relying on standardized protocols, and current implementations typically draw on molecular biomarkers, imaging and sensor-derived functional data. The present study does not operationalize precision rehabilitation in that sense: it uses five generic synthetic scenarios and incorporates no individual-level biological, imaging or functional data. It addresses a narrower and logically prior question — whether a large language model can convert a written clinical description into a structured, scenario-specific rehabilitation program at all — which is a necessary but by no means sufficient condition for any future contribution of such models to precision rehabilitation. The limits of this framing are set out explicitly in the Discussion.

This study aims to generate rehabilitation programs using ChatGPT for sports-related clinical cases and to evaluate them through blinded assessment by two independent sports physiotherapy experts. By combining quantitative rubric scoring with qualitative expert commentary, this study aims to identify the strengths and limitations of LLM-based program generation in sports rehabilitation and to provide actionable insights for future clinical and research applications.

## 2 Materials and methods

### 2.1 Study design

This study used a mixed-methods design to evaluate the quality and clinical applicability of rehabilitation programs generated by ChatGPT for common sports-related injuries. The study combined quantitative expert scoring with qualitative expert commentary to assess the scientific accuracy, structural organization, and clinical relevance of the AI-generated rehabilitation plans. The integration of objective scoring with expert commentary was intended to provide a comprehensive assessment of the usefulness of artificial intelligence in sports rehabilitation planning. The qualitative component is specified as follows. Alongside the numerical ratings, each expert provided free-text commentary on a structured evaluation form. These comments were analysed using deductive qualitative content analysis (20), with an *a priori* coding frame derived from the four rubric domains and supplemented by inductively generated codes for issues not captured by the rubric. Coding was carried out independently by two authors who took no part in the rating, and coding discrepancies were resolved by discussion; because the comment corpus was small, no formal coding-reliability coefficient was calculated, and this is acknowledged in the Limitations.

The research focused on ChatGPT’s ability to generate structured, progressive rehabilitation programs for the different musculoskeletal conditions commonly encountered in sports physiotherapy practice.

### 2.2 Case scenario development

To evaluate ChatGPT’s ability to generate rehabilitation programs for different tissue types and anatomical regions, five clinical case scenarios representing common sports-related musculoskeletal conditions were developed. The cases were designed to reflect realistic clinical presentations frequently encountered in sports physiotherapy practice.

To ensure diversity in rehabilitation strategies, the selected cases involved injuries affecting muscle, ligament, tendon, neural structures, and bone. All cases described recreational athletes aiming for a safe return to sport:

- Hamstring strain (muscle injury)
- Anterior cruciate ligament (ACL) reconstruction rehabilitation (ligament injury)
- Rotator cuff tendinopathy (tendon pathology)
- Lumbar disc herniation with radiculopathy (neural involvement)
- Clavicle fracture rehabilitation after bone union (bone injury)

Each case scenario included standardized patient characteristics - such as age, activity level, symptoms, and functional limitations - to provide sufficient clinical context while maintaining consistency across prompts. All cases described recreational athletes aiming for a safe return to sport, representing a patient population commonly encountered in sports rehabilitation.

### 2.3 Clinical case scenarios

#### 2.3.1 Case 1 — hamstring strain

A 24-year-old male recreational football player sustained a grade II hamstring strain during sprinting (graded according to the Munich consensus statement on the terminology and classification of muscle injuries in sport, corresponding to a type 3B partial muscle tear; on the British Athletics Muscle Injury Classification the described findings correspond approximately to a grade 2b myotendinous lesion) (21, 22). The injury had occurred 2 weeks before assessment. The athlete reported pain during running and difficulty with explosive movements such as sprinting and jumping. Clinical examination revealed localized tenderness over the proximal hamstring region, mild strength deficit on resisted knee flexion, and reduced flexibility of the posterior thigh. The athlete’s primary goal was to safely return to football training and sprinting activities. This case scenario was designed to align with current clinical practice guideline recommendations for the assessment and rehabilitation of hamstring injuries (23). The classification system is stated explicitly because prognosis and expected rehabilitation timelines differ between grading systems, and this affects the standard against which the generated program should be judged.

#### 2.3.2 Case 2 — anterior cruciate ligament (ACL) reconstruction

A 22-year-old female amateur basketball player was undergoing rehabilitation following anterior cruciate ligament reconstruction with a hamstring graft. The surgery had been performed 8 weeks earlier. The patient presented with mild quadriceps weakness, reduced knee flexion strength, and decreased confidence during dynamic movements. The athlete’s goal was to progress gradually toward sport-specific activities and return to basketball. This scenario was constructed to include clinical features consistent with current international consensus statements on return-to-sport criteria after ACL reconstruction (24).

#### 2.3.3 Case 3 — rotator cuff tendinopathy

A 27-year-old male recreational tennis player presented with rotator cuff tendinopathy in the dominant shoulder. Symptoms had persisted for approximately 6 weeks and included shoulder pain during overhead activities and serving motions. Clinical examination revealed mild weakness in shoulder external rotation and abduction, with discomfort on resisted testing. The athlete wished to return to tennis without pain during overhead strokes. This case was designed to include clinical findings consistent with current clinical practice guideline recommendations for the diagnosis, non-surgical management, and rehabilitation of rotator cuff tendinopathy (25).

#### 2.3.4 Case 4 — lumbar disc herniation with radiculopathy

A 29-year-old male recreational runner presented with L4-L5 lumbar disc herniation accompanied by mild radicular symptoms in the right lower extremity. The condition had developed gradually over the past month and was exacerbated by prolonged sitting and running. The patient reported intermittent low back pain and occasional discomfort radiating to the posterior thigh. The rehabilitation goal was to restore spinal stability, reduce symptoms, and enable a safe return to recreational running. This scenario presents a clinical picture consistent with recent systematic review and meta-analysis findings on the effectiveness of exercise therapy for lumbar disc herniation (26).

#### 2.3.5 Case 5 — clavicle fracture rehabilitation after bone union

A 23-year-old male recreational cyclist sustained a midshaft clavicle fracture after a fall from a bicycle. Radiographic examination confirmed successful bone union after 6 weeks of immobilization. The athlete currently reported shoulder stiffness and mild weakness during shoulder elevation. The rehabilitation goal was to restore shoulder mobility, regain strength, and safely return to cycling activities. This case reflects a recovery timeline consistent with recent systematic review and meta-analysis data on return to sport after clavicle fracture (27).

### 2.4 Prompt structure

For each case scenario, a standardized prompt was used to generate a rehabilitation program with ChatGPT. The prompt was designed to guide the model toward producing structured, progressive rehabilitation recommendations.

The instruction used was as follows:

*“Based on the following clinical scenario, design a progressive 6-week sports rehabilitation program. Please include rehabilitation phases, exercise selection, sets and repetitions, progression criteria, and return-to-sport considerations.”*

To provide sufficient context for generating individualized rehabilitation recommendations, the full clinical scenario was included in the prompt.

To ensure consistent formatting and facilitate expert evaluation, ChatGPT was asked to present the rehabilitation program in a predefined table format containing the following columns:

Phase | Week | Exercise | Sets | Repetitions | Progression Criteria | Notes.

A uniform six-week horizon was imposed across all five scenarios for two reasons: to hold the requested output format and length constant so that programs could be compared on an identical rubric, and because a six-week block corresponds to a conventional unit of rehabilitation planning in routine practice. The appropriateness of this constraint is, however, not uniform across the five cases. Six weeks is a clinically congruent block for clavicle fracture rehabilitation after radiographic union, whereas for a patient 8 weeks after ACL reconstruction it represents only one segment of a rehabilitation horizon extending to 6–12 months or longer. Case complexity and prompt–scenario congruence are therefore confounded by design and cannot be separated in the present analysis; this is stated as a limitation and revisited in the Discussion.

### 2.5 AI interaction

ChatGPT-4.1 (OpenAI, San Francisco, USA) was used via its web-based interface with default settings to generate the rehabilitation programs. For each case scenario, the standardized prompt was entered in a separate chat session to minimize contextual bias between responses. Each session was a newly opened chat within a single institutional account; memory, cross-chat history referencing and custom instructions were disabled, no custom GPT was used, and no other account-level personalisation was active. Exactly one generation was obtained per case, and no output was regenerated, re-sampled, selected or discarded. To maintain consistency, no follow-up prompts or clarifications were provided after the initial instruction. The outputs generated by the model were recorded immediately and evaluated without any modification. The AI interactions were conducted in March 2026. Because large language model outputs are stochastic, a single generation per case cannot distinguish a stable model characteristic from one unfavourable sample; repeated generations were not performed, and the consequences of this choice, together with those of model updating and prompt sensitivity, are addressed explicitly in Discussion and Limitations. The verbatim generation prompt is reported in full in Section 2.4 above, and the five unmodified model outputs, together with the completed rating forms, are retained by the author team and are available from the corresponding author on reasonable request, so that the specific generations evaluated here can be inspected and independently re-scored.

### 2.6 Expert evaluation

The rehabilitation programs generated by ChatGPT were evaluated by two independent sports physiotherapy experts, each with more than 20 years of clinical experience in sports rehabilitation. Both experts are actively engaged in clinical practice and have extensive experience in designing rehabilitation programs for athletes. Both raters were independent of the author group: neither is an author of this manuscript, and neither contributed to case scenario development, prompt design, rubric construction, statistical analysis or manuscript preparation. Their involvement was limited to independent scoring and free-text commentary, and they received no information about the study’s expected findings.

The experts evaluated the programs independently, without influencing each other’s assessments. Blinding was partial, and its scope is specified here to remove the ambiguity of the earlier wording. Each rater received the complete clinical scenario together with the unmodified model output, since both are required in order to judge accuracy and clinical applicability at all. What was withheld was: the verbatim wording of the generation prompt, including the imposed six-week horizon and the requested table columns; the identity, version and provider of the model used; the study aims and hypotheses; and the co-rater’s scores and comments. Raters were therefore blinded to the generating conditions and to one another, but not to the clinical scenario.

The rehabilitation programs were assessed using a structured scoring system consisting of four criteria: accuracy, exercise selection, progression, and clinical applicability. Each criterion was scored using a five-point Likert scale ranging from 1 (very poor) to 5 (excellent). The rubric was developed specifically for this study rather than adapted from a previously validated instrument, since no validated tool for appraising AI-generated rehabilitation programs was available at the time of the study. The four domains were derived from components of program quality that recur in existing appraisal frameworks for exercise prescription and in previous expert-evaluation studies of LLM-generated programs (8–10): factual and guideline concordance (accuracy); appropriateness of the selected exercises for the injured tissue and rehabilitation stage (exercise selection); the internal logic and the criteria governing advancement between phases (progression); and the feasibility of implementing the program as written in routine practice (clinical applicability). Content validity was addressed through expert review: the draft rubric, comprising a definition for each domain and anchor descriptors for each point of the five-point scale, was circulated to both raters before scoring commenced, and the wording was revised until both confirmed that the domains were mutually intelligible and non-overlapping. No formal psychometric validation (e.g., content validity index, factor analysis, or testing in an independent sample) was undertaken, and the rubric therefore remains unvalidated; this is acknowledged in the Limitations. The anchor descriptor for each point of the five-point scale, together with the completed rating forms, is available from the corresponding author on reasonable request.

In addition to the quantitative scoring, the experts were asked to provide qualitative comments identifying the strengths, limitations, and potential areas for improvement of the AI-generated rehabilitation programs. Comments were recorded as free text in a dedicated field beneath each domain of the evaluation form, with no word limit and no prompting as to content, so that each rater produced up to four comment entries per case (a maximum of 40 entries across the dataset).

### 2.7 Statistical analysis

Descriptive statistics were calculated for all rubric scores. The mean and standard deviation were computed for each evaluation criterion. Because the pooled mean across all 40 ratings aggregates variance arising simultaneously from cases, criteria and raters, means are additionally reported disaggregated by case and by criterion, each accompanied by a 95% confidence interval. Inter-rater agreement between the two experts was assessed using the intraclass correlation coefficient (ICC) to determine the reliability of the scoring process. The ICC was calculated using a single-measures form based on a two-way random-effects model reflecting absolute agreement (ICC[2,1]) and was interpreted according to the classification criteria proposed by Koo and Li (28) (<0.50 poor, 0.50–0.75 moderate, 0.75–0.90 good, >0.90 excellent) (28). The unit of analysis for the ICC was the individual case × criterion rating unit: five cases × four criteria yielded 20 rating units, each scored by both raters, which is the source of the reported *F*(19,19). Because the four criteria are nested within the same five cases, these 20 units are not strictly independent observations, and the pooled ICC may therefore be somewhat optimistic. To address this, ICC(2,1) was additionally computed separately within each criterion across the five cases, so that each supplementary estimate rests on five independent cases; all ICC estimates are reported with 95% confidence intervals, as recommended by the same source cited for their interpretation (28). Agreement was characterised further by the proportion of exact and within-one-point agreement, linearly and quadratically weighted Cohen’s *κ*, the mean signed between-rater difference with Bland–Altman 95% limits of agreement, and the Wilcoxon signed-rank test for systematic between-rater difference. All statistical analyses were performed using standard statistical software (Python 3.10, NumPy/Pandas/SciPy/pingouin), and statistical significance was set at *p* < 0.05.

## 3 Results

### 3.1 Expert scores by case and criterion

The raw scores assigned by the two expert raters for accuracy, exercise selection, progression, and clinical applicability across the five clinical cases are presented in Table 1. Throughout the Results and in Table 1, paired scores are reported in the format R1 = x, R2 = y, where R1 and R2 denote Rater 1 and Rater 2 respectively; the oblique notation used previously (e.g., “3/4”) has been replaced because it can be misread as a fraction. The overall mean of the 40 ratings (5 cases × 4 criteria × 2 raters) was 3.85 ± 1.21 (95% CI 3.48–4.22) (on a 1–5 scale). This pooled figure combines between-case, between-criterion and between-rater variance and is therefore reported only as a global summary; the disaggregated case- and criterion-level values below carry the interpretable information.

**Table 1:** Expert scores by case and evaluation criterion (R1 = Rater 1, R2 = Rater 2).

Case	Accuracy	Exercise selection	Progression	Clinical applicability	Case Mean ± SD
Case 1 – Hamstring strain	R1 = 3, R2 = 4	R1 = 4, R2 = 4	R1 = 4, R2 = 4	R1 = 3, R2 = 4	3.75 ± 0.46
Case 2 – ACL reconstruction	R1 = 1, R2 = 2	R1 = 1, R2 = 3	R1 = 2, R2 = 3	R1 = 1, R2 = 2	1.88 ± 0.83
Case 3 – Rotator cuff tendinopathy	R1 = 5, R2 = 5	R1 = 5, R2 = 5	R1 = 3, R2 = 4	R1 = 4, R2 = 4	4.38 ± 0.74
Case 4 – Lumbar disc herniation	R1 = 4, R2 = 4	R1 = 5, R2 = 5	R1 = 4, R2 = 4	R1 = 4, R2 = 4	4.25 ± 0.46
Case 5 – Clavicle fracture	R1 = 5, R2 = 5	R1 = 5, R2 = 5	R1 = 5, R2 = 5	R1 = 5, R2 = 5	5.00 ± 0.00
Criterion Mean ± SD	3.80 ± 1.40	4.20 ± 1.32	3.80 ± 0.92	3.60 ± 1.26	3.85 ± 1.21

At the case level, the highest performance was observed for Case 5 (clavicle fracture rehabilitation after bone union), where both experts assigned the maximum score (R1 = 5, R2 = 5) on all four criteria, yielding a mean of 5.00 ± 0.00. In contrast, the lowest performance was recorded for Case 2 (ACL reconstruction) (mean = 1.88 ± 0.83, 95% CI 1.18–2.57), with particularly low scores for accuracy (R1 = 1, R2 = 2) and clinical applicability (R1 = 1, R2 = 2). For descriptive purposes only, case means were assigned to pre-specified bands of the 1–5 scale: low (<2.50), moderate (2.50–3.99), moderate-to-high (4.00–4.74) and high (≥4.75). These bands are descriptive conventions adopted for readability and carry no inferential status. On this basis, Case 3 (rotator cuff tendinopathy, mean = 4.38 ± 0.74, 95% CI 3.75–5.00) and Case 4 (lumbar disc herniation, mean = 4.25 ± 0.46, 95% CI 3.86–4.64) fell in the moderate-to-high band, Case 1 (hamstring strain, mean = 3.75 ± 0.46, 95% CI 3.36–4.14) in the moderate band, Case 5 in the high band and Case 2 in the low band.

### 3.2 Performance by criterion

When examined at the criterion level (Table 2), the highest mean score was obtained for exercise selection (4.20 ± 1.32, 95% CI 3.26–5.14), and the lowest for clinical applicability (3.60 ± 1.26, 95% CI 2.70–4.50). Although accuracy (3.80 ± 1.40, 95% CI 2.80–4.80) and progression (3.80 ± 0.92, 95% CI 3.14–4.46) had similar mean values, accuracy showed the greatest variability across cases, reflecting ChatGPT-4.1’s inconsistent ability to ensure scientific accuracy in certain clinical presentations (e.g., ACL reconstruction). The confidence intervals are wide and overlap substantially across all four criteria; between-criterion differences should therefore be read as descriptive rather than as evidence of differential model performance.

**Table 2:** Descriptive statistics and inter-rater reliability by criterion with 95% confidence intervals.

Criterion	Mean ± SD (*n* = 10)	Min–Max
Accuracy	3.80 ± 1.40 (95% CI 2.80–4.80)	1–5
Exercise selection	4.20 ± 1.32 (95% CI 3.26–5.14)	1–5
Progression	3.80 ± 0.92 (95% CI 3.14–4.46)	2–5
Clinical applicability	3.60 ± 1.26 (95% CI 2.70–4.50)	1–5
Overall	3.85 ± 1.21 (95% CI 3.48–4.22)	1–5

Inter-rater reliability: ICC(2,1) = 0.84 (95% CI 0.52–0.94) (absolute agreement, good level); F(19,19) = 15.32; *p* < 0.001. Unit of analysis: 20 case × criterion rating units, each scored by both raters. Per-criterion ICC(2,1) across the five cases: accuracy 0.91 (0.43–0.99), exercise selection 0.79 (0.07–0.98), progression 0.79 (0.09–0.97), clinical applicability 0.89 (0.35–0.99). Exact agreement 13/20 (65%); within-one-point agreement 19/20 (95%); linearly weighted κ = 0.69; quadratically weighted κ = 0.83; mean difference (R2 − R1) = 0.40 (Bland–Altman 95% limits of agreement −0.77 to +1.57).

### 3.3 Inter-rater reliability

The scoring consistency between the two experts was assessed using a two-way random-effects model (ICC[2,1], absolute agreement). The overall ICC value was 0.84 (95% CI 0.52–0.94) [*F*(19,19) = 15.32; *p* < 0.001], corresponding to a “good” level of reliability according to the classification criteria of Koo and Li (28). The confidence interval extends from the moderate into the excellent range, so with 20 rating units and two raters the point estimate should be treated as provisional. Per-criterion ICC(2,1) values computed across the five cases were 0.91 (95% CI 0.43–0.99) for accuracy, 0.79 (0.07–0.98) for exercise selection, 0.79 (0.09–0.97) for progression and 0.89 (0.35–0.99) for clinical applicability; these intervals are wider still because each estimate rests on only five cases. Complementary agreement indices were: exact agreement 13/20 rating units (65%), within-one-point agreement 19/20 (95%), linearly weighted *κ* = 0.69 and quadratically weighted κ = 0.83. Rater 2 scored on average 0.40 points higher than Rater 1 (SD of the differences 0.60; Bland–Altman 95% limits of agreement −0.77 to +1.57; Wilcoxon signed-rank *p* = 0.011), indicating a small but systematic difference in leniency alongside good relative agreement. Disagreement was confined to 7 of the 20 rating units and was concentrated in the two lowest-scoring cases. The single two-point discrepancy occurred for exercise selection in Case 2 (R1 = 1, R2 = 3); one-point discrepancies occurred for accuracy and for clinical applicability in Case 2 (R1 = 1, R2 = 2 in both), for progression in Case 2 (R1 = 2, R2 = 3), for accuracy and for clinical applicability in Case 1 (R1 = 3, R2 = 4 in both), and for progression in Case 3 (R1 = 3, R2 = 4).

### 3.4 Visual analysis

**Figure 1:**
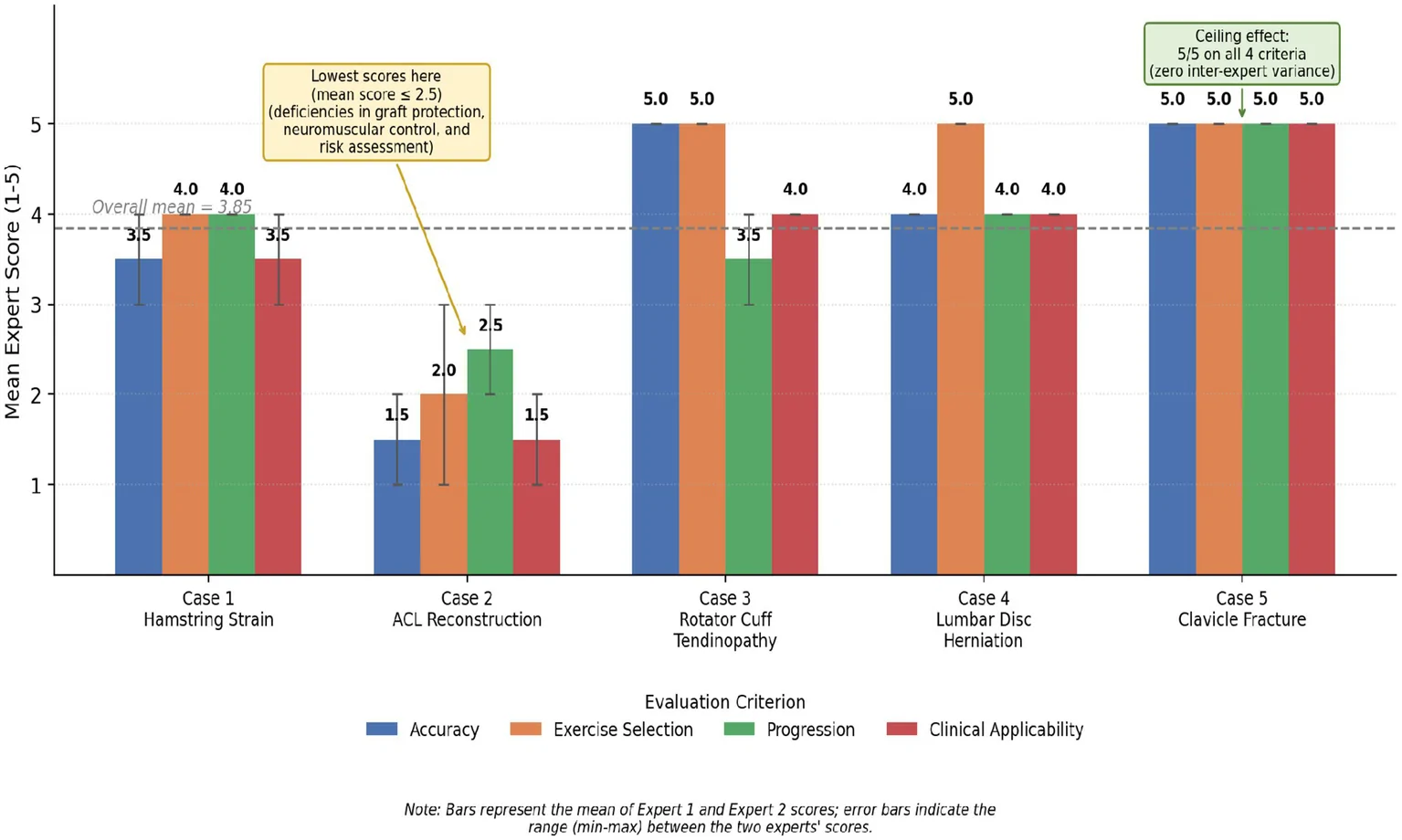
Mean expert scores by case and evaluation criterion (error bars indicate the inter-expert difference).

**Figure 2:**
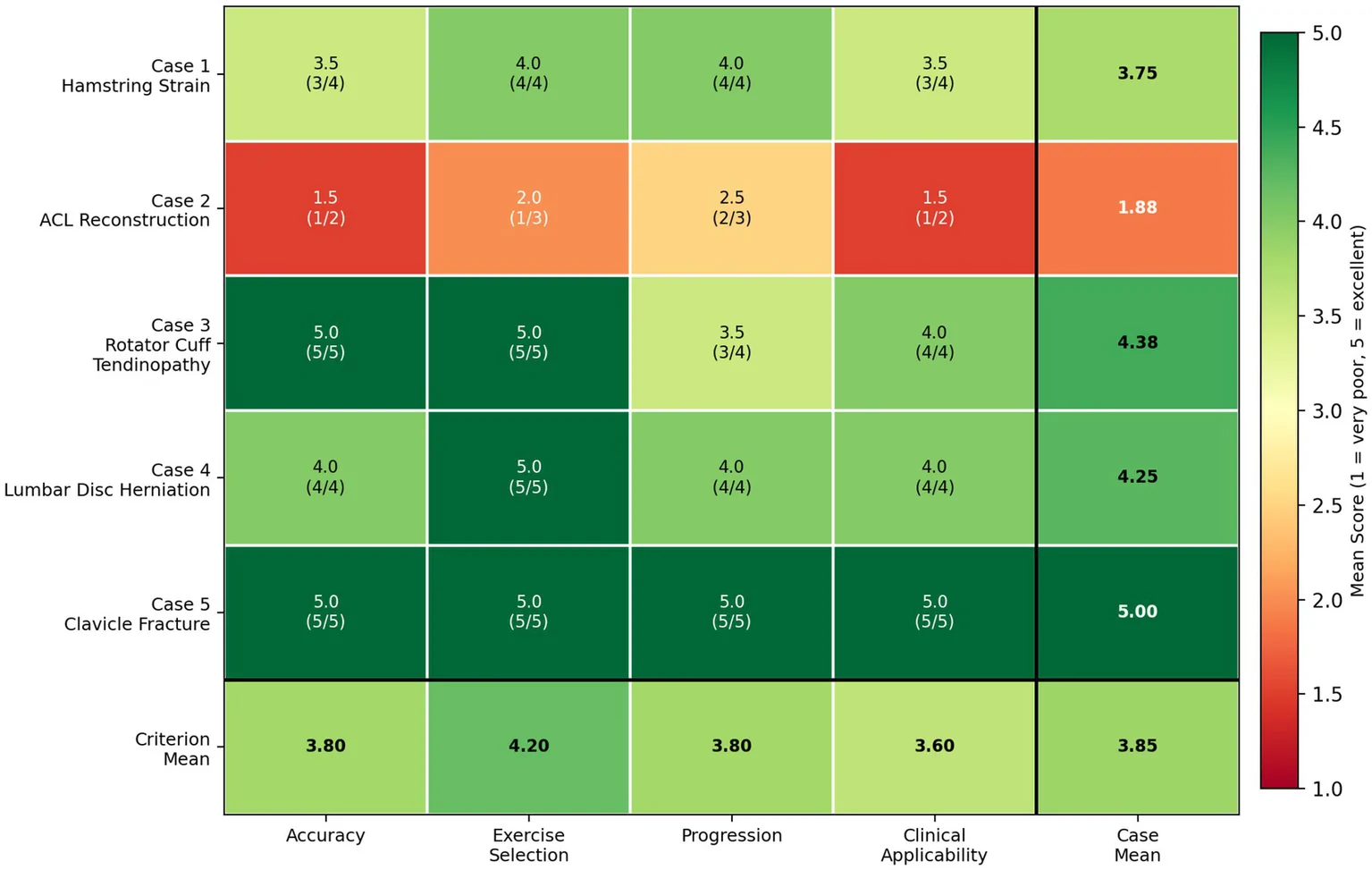
Case × criterion heatmap (mean score; Rater 1 / Rater 2 scores in parentheses).

Figure 1 shows the mean scores for the four criteria for each case, together with the inter-expert score difference (error bars). In Case 2, low scores across all criteria and relatively large inter-expert differences were observed, whereas in Case 5 a ceiling effect (maximum score, zero variance) was evident across all criteria. The heatmap in Figure 2 visually summarizes the score distribution across the case × criterion matrix and clearly shows that the ACL reconstruction case (Case 2) is markedly distinct from the other four cases.

### 3.5 Qualitative expert comments

Both raters returned free-text commentary for every case. Comments were coded as described in Section 2.1, and the synthesis below distinguishes points raised independently by both raters from those raised by one rater only; representative comments are quoted verbatim (translated from Turkish where applicable). The experts’ qualitative evaluations highlighted that ChatGPT-4.1 generally adhered to the requested table format (phase, week, exercise, sets, repetitions, progression criteria, notes) and provided a logical progression structure across phases. In cases with linear and largely protocol-based recovery processes (clavicle fracture, lumbar disc herniation), the program content largely aligned with current rehabilitation principles (gradual restoration of range of motion, progression from isometric to isotonic and functional loading, and pain-guided progression).

In contrast, for the ACL reconstruction case (Case 2), the experts noted that the program did not adequately address the graft-protection timeline (e.g., avoidance of open-chain quadriceps exercises in the early phase) or neuromuscular control and proprioception training, that progression criteria were based on general time intervals rather than objective functional tests (e.g., single-leg hop test, isokinetic strength ratios), and that elements of risk assessment necessary for return-to-sport decision-making were lacking. Similarly, for the hamstring strain case (Case 1), exercise progression was rated as moderate in terms of accuracy and clinical applicability, with eccentric loading dosage and sprint-specific reconditioning phases reported to be insufficiently detailed. Both raters independently raised the absence of graft-protection content and the absence of objective, test-based progression criteria in Case 2, and both independently raised the insufficient specification of eccentric loading dosage in Case 1; the judgement that the Case 2 exercise selection was unsafe as written, rather than merely generic, was raised by one rater only and is the source of the single two-point scoring discrepancy in the dataset. The full set of expert comments, in the original wording, is available from the corresponding author on reasonable request.

## 4 Discussion

This study systematically examined the quality and clinical applicability of rehabilitation programs generated by ChatGPT-4.1 for five different sports injury scenarios through evaluation by two independent experts. The overall mean score (3.85/5) indicated moderate-to-good performance by ChatGPT-4.1, but considerable variability was observed across cases (ranging from 1.88 to 5.00). This finding is consistent with previous literature indicating that LLM performance varies substantially according to the complexity of the clinical scenario (7, 8).

The most striking finding is the performance gap between cases based on linear, largely standardized protocols (clavicle fracture rehabilitation, Case 5) and cases requiring multifactorial, postoperative-staging-sensitive, individualized risk assessment (ACL reconstruction, Case 2). For the clavicle fracture case, ChatGPT-4.1 successfully reproduced a standard shoulder mobilization and strengthening progression following bone union - a protocol that is extensively documented in the literature and depends on relatively few individual variables. In contrast, ACL reconstruction represents a highly individualized decision space requiring the integration of graft type, surgical technique, tissue-healing timelines, neuromuscular control deficits, and evidence-based, test-driven return-to-sport criteria (e.g., limb symmetry index, hop tests). This finding is consistent with previous studies (29) suggesting that, because of the distribution of their training data toward “average” or most commonly represented clinical scenarios, LLMs may show reduced performance in cases involving atypical or high-risk decision points. Similarly, in the comparative evaluation by Genç et al., (9), ChatGPT versions were reported to produce programs aligned with general training principles but to diverge significantly from human experts on individualized parameters such as starting load and target heart-rate range.

The low performance recorded for Case 2 (ACL reconstruction) warrants careful interpretation, and our original account of it has been revised. The prompt requested a six-week program for a patient already 8 weeks past reconstruction; a six-week block spanning approximately weeks 8 to 14 postoperatively is an ordinary segment of ACL rehabilitation and does not, in itself, imply return to sport at 6 weeks. The deficiency identified by the raters lay in the content of the block rather than in its length: the generated program made no reference to graft-protection constraints (for example the avoidance of early open-chain quadriceps loading), contained no objective criteria for advancement, and presented return-to-sport considerations as the terminal element of the six-week block without stating that safe return after ACL reconstruction typically requires 6–12 months or longer and depends on biological tissue healing, recovery of neuromuscular control and the fulfilment of objective functional criteria (e.g., limb symmetry index ≥ 90%, hop test batteries) (24). The claim we now make is therefore narrower than in the original submission: the model did not situate the requested six-week block within the longer postoperative horizon, nor flag that the return-to-sport element it supplied could not be reached within that window. The complete Case 2 output is retained by the author team and is available from the corresponding author on reasonable request, so that this reading can be assessed against the generation itself.

This observation points to a broader tension that large language models face in clinical decision-support contexts: the model reproduced the requested format and timeframe without commenting on how that frame related to the wider recovery trajectory. Deference of this kind to premises embedded in a user’s instruction is consistent with sycophancy — the tendency of models trained with human feedback to conform to user expectations at the expense of accuracy — which has been characterised empirically in the machine-learning literature (30, 31). Considered alongside the overconfidence and hallucination tendencies reported by Kim et al. (29) and Omar et al. (32), this defines a recognisable failure mode for clinical use, with a direct practical implication: structural coherence in an LLM-generated program is not evidence of clinical correctness, and where a prompt embeds a clinically consequential assumption the model is likely to process it as given rather than interrogate it. Evaluation of LLM outputs should accordingly include the model’s capacity to question or qualify clinical premises contained in the prompt — a capacity that could be tested directly by supplying prompts with deliberately unrealistic timeframes or parameters.

The ICC(2,1) value of 0.84 (95% CI 0.52–0.94) indicates a “good” level of inter-rater reliability according to the criteria of Koo and Li (28). This result indicates that the two independent clinicians largely agreed on the overall quality of the ChatGPT-4.1 outputs, and therefore that the findings do not depend on the subjective judgment of a single rater. The width of the interval is itself informative: extending from the moderate into the excellent range, it shows that with 20 rating units and two raters the estimate is imprecise, and the result is best read as compatible with good agreement rather than as establishing it. The fact that the point estimate remained below the “excellent” threshold (>0.90) reflects small but systematic differences between the experts for certain case-criterion combinations (e.g., accuracy and clinical applicability in Case 1, R1 = 3, R2 = 4), consistent with the mean between-rater difference of 0.40 points reported above. Such differences may arise from individual variation in evaluation thresholds related to clinical experience, or from subtle nuances in the interpretation of rubric items, and suggest that the use of more detailed, behaviorally anchored scoring rubrics in future studies could further improve inter-rater consistency.

Our findings are consistent with the study by Safran and Yildirim (8), which reported that ChatGPT shows generally high alignment with clinical practice guidelines in musculoskeletal rehabilitation, but that this alignment is lower for rehabilitation-specific recommendations (weighted kappa = 0.56) than for disease-information questions (weighted kappa = 0.90). Notably, in our study, clinical applicability - the lowest-scoring criterion (3.60 ± 1.26) - supports this pattern: although ChatGPT-4.1 was relatively strong in terms of general exercise selection and structural progression (4.20 ± 1.32), experts remained more cautious about the direct, unmodified applicability of these recommendations in real clinical settings. This is also consistent with the findings of Luo et al. (7), who emphasized that ChatGPT shows promising performance in structured tasks and basic medical guidance, but exhibits inconsistent performance in complex clinical scenarios, limited regional adaptability, and inadequate safety considerations for special populations. A similar pattern of variability has been reported in a recent systematic review in orthopaedic sports surgery showing significant differences in the accuracy, quality, and readability of ChatGPT responses (11), in a study showing variable performance of ChatGPT and Google Gemini compared with clinicians on questions related to vestibular rehabilitation education (33), and in a study showing that responses to patient questions about hip arthroscopy were generally satisfactory but in some cases contained erroneous information (34). The comparative literature in musculoskeletal and orthopaedic medicine converges on the same conclusion from a different direction. Keçeci and Karagöz (17) evaluated ChatGPT-4o and DeepSeek AI against the AAOS clinical practice guideline for clavicle fracture management and found that both systems produced coherent and clinically relevant guideline-based responses — a task profile closely resembling our Case 5, which concerned clavicle fracture rehabilitation and was the only scenario to receive the maximum score from both raters on every criterion. Bayrak et al. (18) compared ChatGPT, Claude and Perplexity across scenario-based questions on septic arthritis and reported that, although all three achieved full marks for accuracy and terminological consistency and did not differ in clinical applicability, they diverged significantly in content depth, in the amount of unnecessary elaboration and in reference support. Karagöz et al. (19) reported comparable divergence between ChatGPT-5 and DeepSeek V3 in artificial intelligence-assisted patient education for foot and ankle disorders. Two implications follow for the present study. First, these findings reinforce the interpretation offered above that LLM performance tracks the degree to which a clinical task is codified: where an authoritative guideline exists and the decision space is narrow, outputs are accurate and consistent, whereas performance degrades in the individualized, multifactorial decision space typified by our Case 2. Second, because the dimensions on which models diverge most in that literature — content depth, unnecessary elaboration and reference support — are precisely the dimensions a single-model design cannot examine, the scores reported here should be read as characterising ChatGPT-4.1 in this task set rather than large language models in general. A common theme across these findings is that LLM performance shows an inconsistent distribution depending on task complexity and clinical context, which supports the case-dependent performance pattern observed in our study.

These findings connect to broader safety concerns regarding LLMs in healthcare: LLMs reason inflexibly and tend toward overconfidence in complex clinical scenarios (29), are susceptible to hallucination in decision-support contexts (32) and can produce unsafe responses to patient-posed medical questions (35). The low accuracy and clinical applicability scores for the ACL reconstruction case can be read as a manifestation of this pattern within sports rehabilitation and support the position — already set out above — that ChatGPT-generated programs for postoperative and high-risk cases should not reach patients without review by a qualified clinician. Established ethical frameworks converge on the same requirement: the transparency-based ethics checklist of Ning et al. (36) recommends systematic reporting of accountability, bias assessment and human oversight when generative AI is integrated into clinical settings, and Tangsrivimol et al. (37) emphasise that efficiency gains must be balanced against hallucination risk. The case-dependent performance differences observed here indicate that LLM outputs require a structured validation process before clinical use.

Our findings suggest that ChatGPT-4.1 may provide value in sports rehabilitation in at least three ways: (1) as a tool for generating initial drafts of programs for low-complexity, protocol-based cases; (2) as a time-saving “screening” or brainstorming tool for clinicians, particularly in busy clinics; and (3) as an educational reference point for students reviewing the principles of structured rehabilitation program design. However, model outputs should never be accepted as an independent clinical decision; rather, they should be positioned as an “initial draft” to be evaluated alongside the clinician’s individual patient assessment, surgical protocols, and evidence-based return-to-sport criteria (4–6). This positioning is consistent with recent reviews addressing the strengths and weaknesses of AI in personalized rehabilitation within a SWOT framework (13), and with conceptual analyses indicating that LLMs can add value to administrative and educational tasks in physiotherapy practice, but cannot replace human oversight in clinical decision-making (14).

Consistent with the framing set out in the Introduction, the present findings do not demonstrate precision rehabilitation, and the two passages have been aligned accordingly. Large language models may in future contribute to individualized program design, but current models cannot incorporate biological markers, tissue-healing dynamics, imaging findings or wearable-derived functional data, and the synthetic scenarios used here contained none of these. The contribution of this study to a precision-medicine agenda is therefore confined to a prior and more modest question — whether structured, scenario-specific programs can be generated at all from a written clinical description — and any stronger claim would exceed the evidence presented.

This study has several limitations. First, and most importantly, the effective sample size is very small. Although 40 individual ratings were analysed, these derive from only five AI-generated programs — one per tissue category — so it is the number of independent clinical scenarios, not the number of ratings, that bounds generalizability. Every case-level conclusion rests on *n* = 1, and the 40 ratings must not be read as 40 independent observations. Second, a single standardized prompt was used. LLM performance is highly dependent on prompt design, so this study evaluates one specific prompting strategy rather than the general capability of ChatGPT-4.1; different phrasing, role framing, stepwise reasoning instructions, or the provision of guideline excerpts within the prompt could plausibly yield materially different programs. Third, only one generation was obtained per case. Because model outputs are stochastic, it cannot be determined whether the low scores for Case 2 reflect a stable characteristic of the model or a single unfavourable sample; repeated generations with distributional reporting would be required to separate these possibilities. Fourth, the six-week program length was fixed by the prompt and applied uniformly to all five scenarios. Because the constraint is clinically congruent for some cases and contestable for others, case complexity and prompt–scenario congruence are confounded and their effects cannot be disentangled in this design. Fifth, the study includes no comparator arm. No clinician-authored program for the same scenarios was scored under the same rubric, so the ratings have no internal reference point, and it remains unknown how expert-written programs would have scored on the same instrument. Sixth, the rubric was developed for this study and has not undergone formal psychometric validation; its content validity rests on expert review rather than on an established instrument, and the resulting scores are therefore not directly comparable with those from other evaluation frameworks. Seventh, the ICC was computed over 20 case × criterion units in which the four criteria are nested within five cases, so the units are not fully independent and the pooled estimate may be optimistic; the per-criterion estimates reported above mitigate but do not resolve this. Eighth, only two expert raters participated. Although agreement was good, two raters provide a narrow basis for judgments of clinical quality, produce wide confidence intervals around every reliability estimate, and cannot represent the variation in practice across settings and countries; a larger, multicentre expert panel would improve both scoring robustness and external validity. Ninth, the five scenarios represent comparatively straightforward rehabilitation pathways. Patients with multiple concurrent injuries, postoperative complications, revision procedures or significant medical comorbidities were not examined, and these are precisely the situations that place the greatest demand on clinical reasoning; the present design therefore probably constitutes a favourable test of the model. Tenth, reproducibility is intrinsically constrained in LLM research. Outputs may differ across repeated sessions under identical prompts, the underlying model is updated by the provider without version-level control by the user, and small changes in prompt wording can alter the output. The findings are therefore tied to ChatGPT-4.1 as accessed through the web interface in March 2026 and may not replicate on a later release; reporting the model version, the verbatim prompt and the raw outputs (available from the corresponding author on reasonable request) is intended to make the study auditable rather than exactly reproducible. Eleventh, no iterative interaction with the model was performed. In routine practice clinicians refine AI-generated output through follow-up questions and additional context, so this single-turn design may underestimate the performance achievable in real-world use. Finally, the evaluation was based on synthetic case scenarios rather than real patients, and on a single model at a single time point; prospective studies using real clinical records and long-term outcome data, and comparative evaluation against other systems (e.g., Gemini, Claude, DeepSeek), are needed before any clinical recommendation can be made.

Future studies should include larger case series, comparisons across multiple LLMs, larger and multicenter expert panels, and iterative clinician-AI interaction protocols. In addition, prospective clinical studies evaluating the impact of LLM-generated programs on real patient outcomes (pain, function, time to return to sport) are critical for the safe integration of this technology into sports rehabilitation practice. The adoption of standardized reporting frameworks, such as the generative AI ethics checklist proposed by Ning et al. (36), in the design of such studies could enhance transparency, reproducibility, and clinical reliability. Comparative designs are a particular priority. Recent orthopaedic work benchmarking ChatGPT-4o against DeepSeek AI on guideline concordance (17), ChatGPT against Claude and Perplexity in a clinical scenario (18), and ChatGPT-5 against DeepSeek V3 in patient education (19) provides a template for extending the present rubric-based approach across models. Equally important are the deliberate inclusion of complex scenarios — polytrauma, revision surgery, postoperative complications and significant comorbidity — repeated generations per scenario to quantify output variability, systematic variation of prompt design to characterise prompt sensitivity, and multi-turn clinician-AI interaction protocols that reflect how these tools are actually used.

## 5 Conclusion

This mixed-methods study demonstrated that ChatGPT-4.1 can generate structured rehabilitation programs of moderate-to-good quality (overall mean 3.85/5, 95% CI 3.48–4.22) for five different sports-related clinical scenarios, with good inter-rater reliability (ICC = 0.84, 95% CI 0.52–0.94). Performance varied with case complexity: the highest ratings were obtained for the linear, protocol-based scenario (clavicle fracture rehabilitation after union) and the lowest for the scenario requiring multifactorial postoperative individualization (ACL reconstruction). Because each tissue category is represented by a single scenario (*n* = 1), these are observations about five specific generated programs rather than established properties of the model, and the direction of the difference — not its magnitude — is what the present data support. The findings are consistent with the view that ChatGPT may serve as a complementary, clinician-supervised decision-support tool in sports rehabilitation, but should not be used as an independent source for complex or high-risk clinical decisions. Because the six-week program length was imposed by the prompt on all scenarios, case complexity and prompt–scenario congruence remain confounded, and this qualifies the comparison between cases. Further research with larger case series, multiple prompting strategies, multi-model comparison, larger expert panels and patient-centred outcome data is needed to guide responsible clinical integration. As a research outlook rather than an implication of the present findings, future AI-assisted rehabilitation systems that integrate wearable sensor data, imaging biomarkers and molecular profiling may eventually support a precision-medicine framework for individualized rehabilitation planning; the present study, which used generic synthetic scenarios and no individual-level biological data, does not itself provide evidence for that prospect.

## Data Availability

The original contributions presented in the study are included in the article/Supplementary material, further inquiries can be directed to the corresponding authors.
