# Supplementary material for: ChatGPT-generated rehabilitation programs in sports physiotherapy: an expert evaluation and a mixed-methods study of clinical applicability
Source: Front Med (Lausanne). 2026 Aug 26;13:1935702. doi: 10.3389/fmed.2026.1935702 (PMC13553226; doi:10.3389/fmed.2026.1935702)
Supplement: Supplementary file 1 [file Supplementary_file_1.DOCX]

***Supplementary Material***

**Table 1. Expert scores by case and evaluation criterion (R1 = Rater 1, R2 = Rater 2)**

| **Case** | **Accuracy** | **Exercise Selection** | **Progression** | **Clinical Applicability** | **Case Mean ± SD** |
| --- | --- | --- | --- | --- | --- |
| Case 1 – Hamstring strain | R1 = 3, R2 = 4 | R1 = 4, R2 = 4 | R1 = 4, R2 = 4 | R1 = 3, R2 = 4 | 3.75 ± 0.46 |
| Case 2 – ACL reconstruction | R1 = 1, R2 = 2 | R1 = 1, R2 = 3 | R1 = 2, R2 = 3 | R1 = 1, R2 = 2 | 1.88 ± 0.83 |
| Case 3 – Rotator cuff tendinopathy | R1 = 5, R2 = 5 | R1 = 5, R2 = 5 | R1 = 3, R2 = 4 | R1 = 4, R2 = 4 | 4.38 ± 0.74 |
| Case 4 – Lumbar disc herniation | R1 = 4, R2 = 4 | R1 = 5, R2 = 5 | R1 = 4, R2 = 4 | R1 = 4, R2 = 4 | 4.25 ± 0.46 |
| Case 5 – Clavicle fracture | R1 = 5, R2 = 5 | R1 = 5, R2 = 5 | R1 = 5, R2 = 5 | R1 = 5, R2 = 5 | 5.00 ± 0.00 |
| **Criterion Mean ± SD** | **3.80 ± 1.40** | **4.20 ± 1.32** | **3.80 ± 0.92** | **3.60 ± 1.26** | **3.85 ± 1.21** |

**Table 2. Descriptive statistics and inter-rater reliability by criterion with 95% confidence intervals**

| **Criterion** | **Mean ± SD (n=10)** | **Min–Max** |
| --- | --- | --- |
| Accuracy | 3.80 ± 1.40 (95% CI 2.80–4.80) | 1–5 |
| Exercise Selection | 4.20 ± 1.32 (95% CI 3.26–5.14) | 1–5 |
| Progression | 3.80 ± 0.92 (95% CI 3.14–4.46) | 2–5 |
| Clinical Applicability | 3.60 ± 1.26 (95% CI 2.70–4.50) | 1–5 |
| **Overall** | **3.85 ± 1.21 (95% CI 3.48–4.22)** | **1–5** |

**Inter-rater reliability: ICC(2,1) = 0.84 (95% CI 0.52–0.94) (absolute agreement, good level); F(19,19) = 15.32; p < 0.001. Unit of analysis: 20 case × criterion rating units, each scored by both raters. Per-criterion ICC(2,1) across the five cases: accuracy 0.91 (0.43–0.99), exercise selection 0.79 (0.07–0.98), progression 0.79 (0.09–0.97), clinical applicability 0.89 (0.35–0.99). Exact agreement 13/20 (65%); within-one-point agreement 19/20 (95%); linearly weighted κ = 0.69; quadratically weighted κ = 0.83; mean difference (R2 − R1) = 0.40 (Bland–Altman 95% limits of agreement −0.77 to +1.57).**

**Figure 1. Mean expert scores by case and evaluation criterion (error bars indicate the inter-expert difference)**

**Figure 2. Case × criterion heatmap (mean score; Rater 1 / Rater 2 scores in parentheses)**
